# A Comprehensive and Bias-Free Machine Learning Approach for Risk Prediction of Preeclampsia with Severe Features in a Nulliparous Study Cohort

**DOI:** 10.21203/rs.3.rs-2635419/v1

**Published:** 2023-04-10

**Authors:** Yun Lin, Daniel MALLIA, Andrea CLARK-SEVILLA, Adam CATTO, Alisa LESHCHENKO, Qi YAN, David Haas, Ronald WAPNER, Itsik PE'ER, Anita RAJA, Ansaf SALLEB-AOUISSI

**Affiliations:** Columbia University; CUNY Hunter College; Columbia University; CUNY Hunter College; CUNY Hunter College; Columbia University; Indiana University School of Medicine; Columbia University; Columbia University; CUNY Hunter College; Columbia University

## Abstract

**Objective:**

Preeclampsia is one of the leading causes of maternal morbidity, with consequences during and after pregnancy. Because of its diverse clinical presentation, preeclampsia is an adverse pregnancy outcome that is uniquely challenging to predict and manage. In this paper, we developed machine learning models that predict the onset of preeclampsia with severe features or eclampsia at discrete time points in a nulliparous pregnant study cohort.

**Materials and Methods:**

The prospective study cohort to which we applied machine learning is the Nulliparous Pregnancy Outcomes Study: Monitoring Mothers-to-be (nuMoM2b) study, which contains information from eight clinical sites across the US. Maternal serum samples were collected for 1,857 individuals between the first and second trimesters. These patients with serum samples collected are selected as the final cohort.

**Results:**

Our prediction models achieved an AUROC of 0.72 (95% CI, 0.69–0.76), 0.75 (95% CI, 0.71–0.79), and 0.77 (95% CI, 0.74–0.80), respectively, for the three visits. Our initial models were biased toward non-Hispanic black participants with a high predictive equality ratio of 1.31. We corrected this bias and reduced this ratio to 1.14. The top features stress the importance of using several tests, particularly for biomarkers and ultrasound measurements. Placental analytes were strong predictors for screening for the early onset of preeclampsia with severe features in the first two trimesters.

**Conclusion:**

Experiments suggest that it is possible to create racial bias-free early screening models to predict the patients at risk of developing preeclampsia with severe features or eclampsia nulliparous pregnant study cohort.

## Introduction

Preeclampsia (PE) is one of the leading causes of maternal morbidity, with consequences during and after pregnancy[1]. Ensuring optimal patient outcomes requires robust prediction models for PE risk, emphasizing early detection. However, PE poses significant diagnostic and prognostic difficulties given its variable presentations in terms of clinical indications, speed of development, and timing, as well as its unknown causes. PE might evolve slowly and remain mild or quickly present severe complications leading to what is known as PE with severe features (sPE)[1]. Moreover, there are two sub-categories: early onset PE requiring delivery before 34 weeks and late onset after that. While the early onset of PE is associated with a higher incidence of adverse pregnancy outcomes, understanding the relationship between the early and late onset of PE has proven challenging[2, 3]. Some researchers treat them as distinct but work by Poon et al.[2] treats the condition as a spectrum, best represented by a survival time model. Beyond this, the presence of seizures that cannot be attributed to any other underlying condition in a patient diagnosed with PE would be categorized as Eclampsia (E)[1].

Though a complete understanding of PE still needs to be discovered, rich literature exists on risk factors for and indicators of PE. Biochemical and biophysical markers can have an added benefit for screening for PE when combined with clinical characteristics taken from medical history, demographics, clinical measurements, etc[2, 4, 5, 6, 7]. Research[2, 8, 9, 10] has suggested placental growth factor (PlGF), soluble Flt-1 (sFlt-1), pregnancy-associated plasma protein A (PAPP-A), and ultrasound measurements as clinical factors that are significant in signaling an increase in the risk of PE.

Applying this significant volume of knowledge to prediction is pertinent. This study aims to build bias-free machine learning classifiers at various discrete points in pregnancy that combine well-known risk factors for and indicators of sPE and E, which can help screen for cases early in pregnancy in a nulliparous study cohort. While many other studies have focused on predicting preeclampsia in a general population, our study focuses solely on nulliparous patients, making the prediction tasks much more difficult since no prior obstetrical history information is available.

## Materials And Methods

### Study population

**Figure 1:**
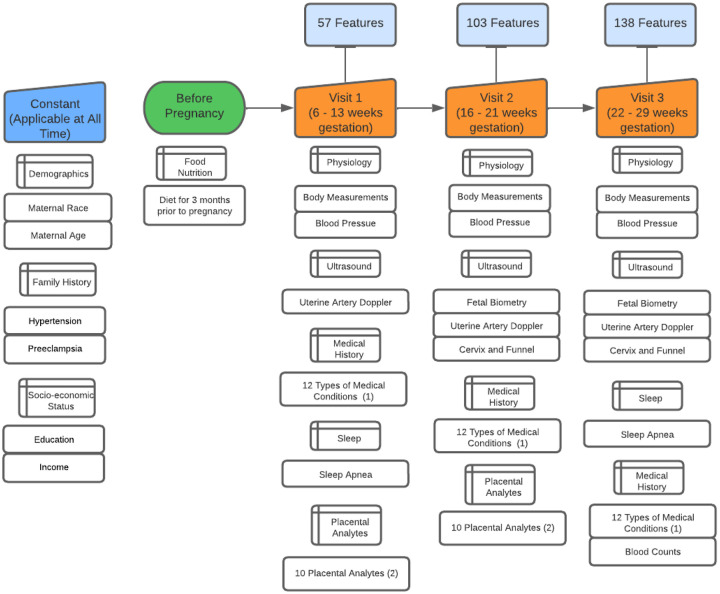
Data process timeline This figure shows the gestational weeks at each visit. For each visit, the number of features at that visit is listed and the category of new feature included is also shown. ^1^ Specific medical condition can be found in Table Supplement 5 ^2^ Specific placental analytes can be found in Table Supplement 3

**Figure 2:**
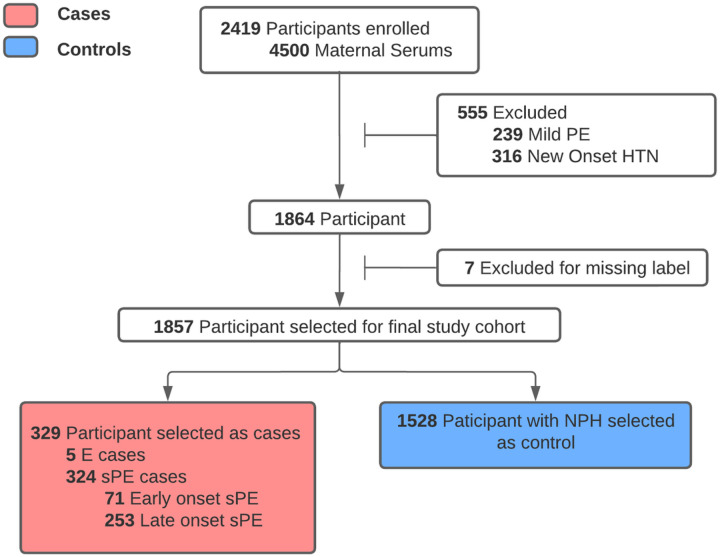
Final study cohort selection process. Out of the participants from the placental analytes sub-study, we excluded participants with conditions such as chronic hypertension, mild preeclampsia, and missing label for preeclampsia to focus on the participants that are most at risk.

The prospective cohort we considered is the Nulliparous Pregnancy Outcomes Study: Monitoring Mothers-to-be (nuMoM2b)[11], which contains information from eight clinical sites across the US between October 2010 and May 2014. Participants gave written informed consent, and institutional review board approval was obtained at all sites. Maternal race was self-reported by participants. The study contains a wide array of information collected for nulliparous participants across four visits, three corresponding roughly to the three trimesters (V1-V3). Ultrasound information was collected at the three visits. All personnel performing ultrasound examinations on patients underwent an ultrasound credentialing process. At V1 and V2, maternal serum was collected, enabling a limited follow-up nuMoM2b sub-study to understand the relationship between placental analytes and a set of adverse pregnancy outcomes (APOs). The multiple of median (MoM) values of the placental analytes were calculated and used as an input to our model. Figure 1 describes in detail the number and categories of features selected, and Fig. 2 contains a flowchart of the final study cohort selection process. For the specific features included in our prediction model, please refer to supplement Tables 1–5. Information from the prior visits is also incorporated into the V2 and V3 prediction models. Therefore, the prediction model for V2 was trained on information from V1 and V2. The prediction model for V3 was trained on data collected from V1, V2, and V3. For V1, 57 features were used to train the model, 103 for V2, and 138 for V3.

To focus on those most at risk, we selected probands with severe PE (sPE). Those with mild preeclampsia, superimposed preeclampsia, and new onset hypertension were excluded. There are no cases of fetal demise at < 20 weeks in the final study cohort. We preserved 36 instances of stillbirth, all of which belonged to the no pregnancy-related hypertension (NPH) category.

### Study outcome

The labeling of sPE was according to the labeling in the nuMoM2b study. Supplement Fig. 1 contains a flowchart indicating the study diagnostic criteria for sPE. The nuMoM2b dataset also contained labels in accordance with the ACOG criteria published in 2013. Initial testing of the proposed pipeline with this ACOG labeling indicated results very similar to that achieved with the nuMoM2b criteria.

### PEPrML pipeline

Our **P**re**E**clampsia **Pr**edictor with **M**achine **L**earning (PEPrML) pipeline produces machine learning-capable models that are explainable and trustworthy. Classifiers to predict sPE + E versus NPH and early sPE versus late sPE + E were modeled for every visit. Categorical features were one-hot encoded. We experimented with multiple, KNN, MICE, and mean imputation for other continuous features and found that all methods produce similar results. The results of the mean imputation were reported. We used a cross-validation strategy that uses 60-20-20 percent train, validation, and test splits, respectively, with 100 different train-val-test splits. The results of the test sets were reported. We balanced the ratio of control versus cases by undersampling in the training and test sets, as this introduces less overfitting, leads to a faster training time, and avoids an over-inflated Area Under the ROC curve (AUC). Therefore, 0.5 was selected as the test positivity cut-off for calculating sensitivity, specificity, positive predictive value (PPV), and negative predictive value (NPV). This process is described in detail in Fig. 3 in Supplement. We experimented with logistic regression (LR), support vector machines (SVM), random forest (RF), and extreme Gradient Boosting (XGBoost)[12]. For RF and XGBoost, we extracted the interpretable feature importance rankings, identifying the top factors to generate partial dependence plots (PDPs)[13]. Two ensemble methods (RF and XGBoost) were chosen as classifiers specifically because they are more robust to noise and overfitting, exhibiting a double descent risk curve[14]. In the supplement material, we provide a detailed analysis of this phenomenon. We use Partial Dependence Plots (PDPs) to display the marginal effect that features of interest have on the predicted outcome of a given model, allowing us to advance our understanding of the outcome. For the RF model, we calculated the Equal Opportunity Ratio (EOR)[15], Predictive Parity Ratio (PPR)[16], Predictive Equality Ratio (PER)[16], Accuracy Equality Ratio (AER)[17], and Statistical Parity Ratio (SPR)[17]. We mitigated the race-based biases using Ceteris Paribus Cutoff Plot. For details, please refer to the Supplement.

### Software packages

We developed our pipeline in Python 3. Instructions about how to run the experiments are provided in the Github repository. We also conducted bias mitigation experiments using the Dalex packaged[18]. Dataset balancing was done using the imbalanced-learn package. The model used to generate our results was trained using the XGBoost package.

The underlying code for this study is available in PRAISE-Lab repository and can be accessed via this link: https://github.com/PRAISE-Lab-Repository/PEPrML.git

#### Ethical Approval

Human subjects approval for this study, titled "SCH: Prediction of Preterm Birth in Nulliparous Women", was obtained following review by Columbia University Human Subjects Institutional Review Board, and the City University of New York CUNY Institutional Review Board.

#### Data Availability

The data that support the findings of this study are available from NIH Data and Specimen Hub, but restrictions apply to the availability of these data, which were used under licence for the current study, and so are not publicly available. Data are however available from the authors upon reasonable request and with permission of NIH Data and Specimen Hub.

## Results

### Study population characteristics

1,857 participants were selected as the final study cohort. Among these, 5 developed E and 324 developed sPE, of which 71 (~22%) were early onset (<34 weeks), and 253 (~78%) were late onset. The remaining 1,528 patients were NPH (Figure 2). Participants had a median age of 27 and IQR of 9; 3.3% were Asian, 17.6% were Hispanic, 57.5 % non-Hispanic white, 15.9% non-Hispanic black, and 5.7% were of other races or multiracial.

Some significant characteristics (P<0.001) among the sPE+E participants versus NPH include a higher mean value for body mass index (BMI) (27 kg/m2 vs. 24 kg/m2), systolic blood pressure (SBP) (112 mmHg vs. 108 mmHg), diastolic blood pressure (70 mmHg vs. 66 mmHg), a lower mean value for PlGF (0.92 vs. 1.00) and PAPP-A (0.86 vs. 1.00) at V1. Some significant characteristics (P<0.001) among the early sPE participants versus late sPE+E include a lower mean value for PlGF (0.73 vs. 0.98). For a detailed summary of statistics of all features, please refer to supplement Tables 1 - 5.

### Model performance

A summary of performance results for sPE+E versus NPH can be found in Figure 4. Results in Figure 4.a indicate that predictive capabilities increase with gestational age. RF models achieved an AUC of 0.72 (95% CI, 0.69–0.76) at V1, 0.75 (95% CI, 0.71–0.79) at V2, and 0.77 (95% CI, 0.74–0.80) at V3. Welch’s t-test was conducted for each pair of classifiers. RF model performance is significantly different (<0.001) for all visits compared to LR and SVM, while only significantly different to XGboost at V1. Detailed measures for RF and other comparison methods can be found in Table 1. Further performance breakdown is offered in Table 2 and Supplement Table 6, which summarize results for predicting early and late onset, versus NPH, respectively.

The model's predictive power for early onset preeclampsia is higher than for late onset, as demonstrated by the two tables. Across the board, all metrics have higher values, but the variance is also higher for these values, most likely due to the smaller set of cases with early onset sPE. We modeled classifiers to directly predict early sPE vs. late sPE+ E to understand better what enabled this performance. A summary of performance results for early sPE vs. late sPE+E can be found in Figure 4. Again, performance increased with gestational age, and RF models performed the best, obtaining an AUC of 0.64 (95% CI, 0.53 – 0.75) at V1, 0.76 (95% CI, 0.68 – 0.82) at V2, and 0.83 (95% CI, 0.75 – 0.91) at V3. Detailed performance measures for RF and other comparison methods can be found in Table 3.

### Interpreting sPE+E vs NPH model

**Figure 3:**
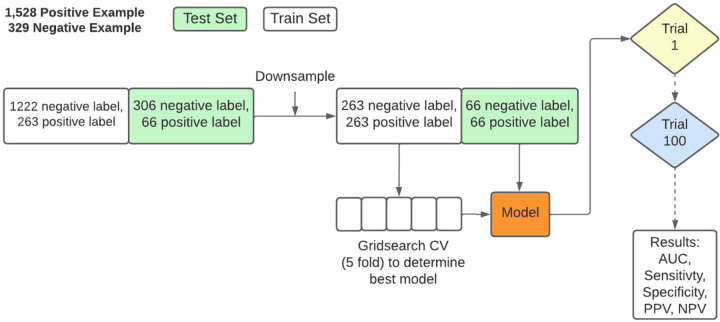
The training process of PEPrML pipeline. Samples were balanced for train and test sets. 5-fold grid search cross-validation was used to select the hyperparameters for each trial. We repeated 100 trials and recorded the results.

The feature importance lists for V1, V2, and V3, where the prediction task is prognosis, are given in supplement Figure 2, Figure 5.a, and supplement Figure 3, respectively, enabling a better understanding of the key features that contribute to the RF and XGBoost decision processes. For V1, the top 5 features are BMI, mean arterial pressure (MAP), SBR waist circumference, and endoglin. For V2, the top five features are BMI, PlGF (V2), MAP (V2, V1), and SBP (V2, V1).

**Figure 4:**
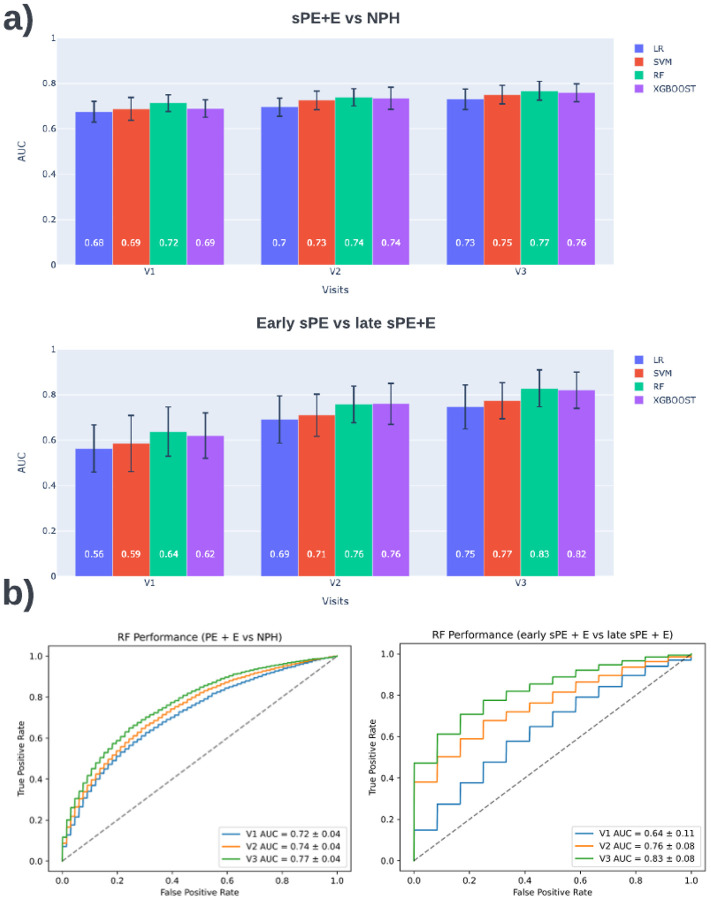
sPE+E vs NPH and early sPE vs late sPE+E model performance. a) Average AUC for 100 trials per visit for 4 classifiers. b) RF classifier has best performance across visits for both comparisons. The ROC curve demonstrated the tradeoff between the true positive rate versus false positive rate. This summarizes the results for 100 trials.

The PDP for BMI shown in Figure 5.c indicates a risk increase in sPE+E at around 22.41 and at the peaks at 35 . We see a substantial increase in the risk of sPE+E with a systolic reading of 110 mmHg or higher, and by Visit 2, this number drops to 102 mmHg (supplement Figure 4.a). The diastolic reading did not exhibit such a pronounced increase in the risk of sPE+E, but we did observe a slight increase above 78 mmHg. Looking at the MAP at Visit 1, supplement Figure 4.b, we see an increase in risk at 82.67 mmHg. There is a sharp increase in the predicted risk for sPE+E observed in the PDP for PlGF at Visit 1 for MoM measurements less than 1.5.

### Racial Fairness in sPE+E vs NPH model

Our model for predicting sPE+E vs. NPH is biased mainly against Black participants. Using the White race as the reference race, we identified that the predictive equality ratio for Black participants (1.31) is high, according to the four-fifths rule.

**Figure 6:**
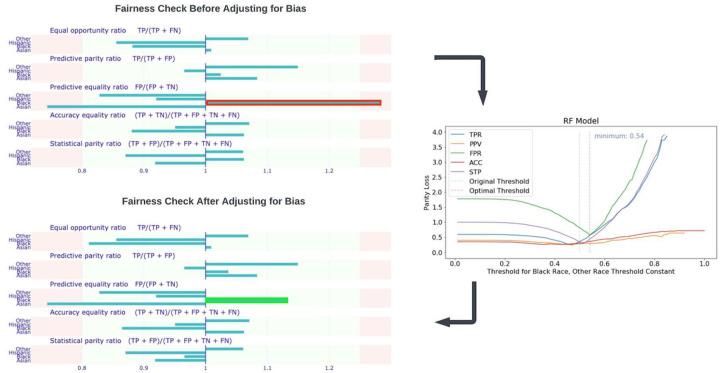
Fairness check for sPE+E vs NPH mode The threshold set based on the four-fifth rule are 0.8 and 1.25. *Ceribus Paribus* plot was used to adjust prediction threshold for the Black population.

To address this problem, we created a *ceteris paribus* Cutoff plot of the parity loss for the Black subpopulation to determine the optimal confidence threshold for prediction. Adjusting the threshold accordingly mitigated the over-prediction of PE occurrence by our model for Black participants, reducing the predictive equality ratio for Black participants from 1.31 to 1.14 (Figure 6).

## Discussion

The results presented here demonstrate that it is possible to learn RF models with superior, well-rounded performance for early prediction of preeclampsia at multiple time points throughout pregnancy, with minimal preprocessing of data, feature engineering, or feature selection. Exhibiting a relatively balanced score for PPV and Sensitivity, RF increases performance by all metrics at each new visit as more information becomes available. The feature importance plots confirm existing knowledge about known predictive features such as blood pressure, uterine artery blood flow, and placental analytes and identify features not commonly referenced in the prediction literature, such as Endoglin, Cholesterol, and Inhibin A. Review of RF fairness metrics indicated a correctable bias against Black participants.

Our study confirmed that blood pressure and placental analytes were significant in predicting PE across study visits[19,20,21]. The results of our statistical tests deviate from other works[2,10,22] in that risk factors such as maternal age, race, sleep apnea, and family history of PE were not significant. Socio-economic status did not contribute to the prediction of preeclampsia in our study cohort, as suggested by other studies such as Arechvo et al[23]. Thus, care must be taken in comparing the model performance presented here for the nuMoM2b dataset with other studies, given that the nuMoM2b dataset characterizes demographically diverse nulliparous mothers with unknown risk for PE at the time of first prediction while the target label is strictly focused on sPE+E criteria.

Our selected predictors in the first trimester of pregnancy are like those used by previously published competing risk models from Akolekar et al., Poon et a I., and O’Gorman et al.[24, 25, 26], but our study contains more features and focuses solely on a nulliparous study cohort. To compare our results to these two prior studies, we reconstructed their experiment using our nulliparous cohort and features from V1. We found that our model yielded better outcomes across the board. In Table 4, our model performance, on average, has a 3-4% higher AUC. While Poon et al.[24] report a 91% AUC for preterm PE and 78% AUC for predicting term PE just by utilizing features such as maternal risk factors, MAP, PlGF, uterine artery pulsatility index, and PAPP-A, we did not observe this high AUC in our prediction model. This might be attributed to the fact that our prediction task focuses on PE with severe features for nulliparous women only, which makes the prediction tasks much more difficult.

Ensemble methods, specifically RF and XGBoost[27], are the top performers in our study. Other studies have shown ensemble methods to have a strong predictive power for preeclampsia [28,29,30]. This may be due to the ensemble nature and the ability of the underlying model, decision trees, to capture some of the subtle distinctions between the varied and poorly understood subgroups of preeclampsia patients[31]. The PDP for BMI, a well-known risk factor for PE, shown in Figure 5.c indicates a risk increase in PE around 22.41 and at the peaks at 35 . One possible rationale is that the effect of magnesium circulation is reduced when the BMI is at 35, since a good magnesium circulation can significant reduce the risk of eclampsia or convulsions[32]. Furthermore, PDPs for various placental analytes indicate that a decreased level of PlGF during the first and second trimesters precede the onset of PE[2,33,34]. Agrawal et al.[35] found that the predictive value was highest for PlGF levels between 80 and 120 pg/mL, which coincides with the sharp increase in the predictive risk for PE observed in the PDP for PlGF at Visit 1 for measurements less than 100 pg/mL. MacDonald et al.[36] suggested a sFlt-1:PlGF ratio > 33.4 which agrees with our PDP in supplement Figure 5. Levine et al.[37] found that endoglin levels at 25 through 28 weeks of gestation were significantly higher (8.5 ng/mL) in term PE patients. We observe this same cutoff value in the PDP in supplement Figure 4.c, which shows a pronounced increase in the risk of PE at around 9 ng/mL at V1, albeit occurring much earlier, at 6-13 weeks of gestation. Analytes such as PlGF, unlike blood pressure, were consistently important across the sPE+E vs. NPH model and the early vs. late model (Figure 5), indicating their predictive power, particularly their ability to *rule out* early onset[4,27].

### Implications

This study demonstrates the utility of early and multiple time points screening for PE. It shows that early blood pressure measurement can be a proxy for the risk of high blood pressure later in pregnancy. Also, information about placental analytes, which can be gathered at a reasonable cost tradeoff between assessment and hospitalization[4], allows predictions that enormously surpass the accuracy of a model based only on ACOG guidelines[38]. Further validation is required for the proposed separate models for multiple time points to ensure prediction consistency: a patient identified as high risk early in pregnancy should not be deemed low risk later without sufficient explanation. Also, identifying women at increased risk in the first trimester allows for timely prophylaxis with low-dose aspirin, which is highly effective in preventing preterm disease[39].

Fairness metrics and analysis of causes for biases should become standard practice in model validation. We hypothesize that the limited sample size may have caused the bias against the Black participants skewed disproportionately towards White participants and the potentially inappropriate higher representation of the Black population among the sPE+E class than the NPH class (20.9% vs. 13.8%, respectively). However, after correcting for this imbalance, the bias still persisted. We then hypothesize that this bias might come from a difference in the distribution of values for the top placental analytes, as suggested in another study[40]. We did observe significant differences in the distribution of top predictive features (P<0.001), such as BMI and PLGF (V1, V2). Due to the correlation between some top features, we cannot simply normalize each by race. Therefore, adjusting the predictive threshold for the Black population is still an efficient way to reduce bias. While the cost of a false negative diagnosis for maternal and fetal health is very high, the stress, fees, and possibly inappropriate treatment of a false positive should not be ignored.

Distinguishing between sPE+E and NPH is critical, but the binary labels pose a challenge. The former group undoubtedly contains different subgroups and phenotypes of preeclampsia, and learning to make these distinctions will have the dual benefit of enhancing our understanding of preeclampsia and allowing for better predictive performance. Thus, moving beyond the initial literature-inspired feature set to a broader set of features will be the target of future work. Furthermore, temporal features capturing change between clinical measurements at different visits will be investigated, as this may enhance prediction quality at the second and third time points[28]. This would enable more timely monitoring and treatment of late onset preeclampsia. A more significant departure will involve re-framing the prediction task. Compelling arguments have been made that preeclampsia is best interpreted as a syndrome rather than a disease[27,41]. Label difficulties have led at least one study of short term preeclampsia screening to focus on a label that consists of the presence, or not, of at least one of multiple maternal or adverse fetal outcomes[27].

### Limitations

A set of features identified in the related medical literature was employed for this initial study, but this can be expanded without issue. Using the nuMoM2b data represents an exciting opportunity to learn from a sizable sample of U.S. mothers that is more diverse than other similar studies and that has been captured in a longitudinal study with a considerable number of features[3,27,42]. The occurrence rate of PE in this study was consistent with reported rates[4,43]. However, this meant that even with such a sizable sample, the analysis was limited to more than a couple of hundred sPE+E cases. The sub-study also had limitations: analytes were only available for V1 and V2. Our study only applies to the nulliparous population within the US. Therefore, our models do not take previous obstetric history into account.

One noticeable limitation of the study is the limited cases of existing medical conditions in participants of the placental analytes sub-study. This low presence can cause the model to attribute less importance to these risk factors, while these could be crucial in clinical practice. Lastly, our study only focuses on comparing patients with sPE+E and NPH, without addressing those patients who developed PE with mild features, or only hypertension.

## Conclusion

Our experiments suggest that it is important and possible to create screening models to predict the participants at risk of developing preeclampsia with severe features and eclampsia for a nulliparous study cohort. The top features stress the importance of using several tests, in particular tests for biomarkers and ultrasound measurements. The models could potentially be used as a screening tool as early as 6-13 weeks gestation to help clinicians screen for and identify participants who may subsequently develop preeclampsia, confirming the cases they suspect or identifying unsuspected cases. The proposed approach is easily adaptable to address any adverse pregnancy outcome with fairness.

## Figures and Tables

**Figure 5:** Interpreting machine learning model for sPE+E vs NPH and early sPE vs late sPE+E a) V2 features importance for sPE+E vs NPH model, b) V2 feature importance for early sPE vs late sPE+E, c - d) PDP for BMI and PlGF based on model build for sPE+E vs NPH

**Table 1:** Detailed summary of sPE+E vs NPH model performance per visit for four classifiers.

Model	Visits	AUC	Sensitivity (TP/ TP+FN)	Specificity (TN/ FP + TN)	PPY (TP/ TP +FP)	NPV (TN/FN+TN)
LR	V1	0.68 ± 0.05	0.59 ± 0.06	0.67 ± 0.06	0.64 ± 0.05	0.62 ± 0.04
	V2	0.70 ± 0.04	0.63 ± 0.05	0.67 ± 0.05	0.65 ± 0.04	0.64 ± 0.03
	V3	0.73 ± 0.04	0.65 ± 0.07	0.69 ± 0.05	0.68 ± 0.04	0.67 ± 0.05
SVM	V1	0.69 ± 0.05	0.57 ± 0.06	0.70 ± 0.06	0.66 ± 0.05	0.62 ± 0.04
	V2	0.73 ± 0.04	0.59 ± 0.06	0.74 ± 0.05	0.70 ± 0.05	0.65 ± 0.04
	V3	0.75 ± 0.04	0.60 ± 0.06	0.77 ± 0.05	0.72 ± 0.05	0.66 ± 0.03
RF	V1	0.72 ± 0.04	0.64 ± 0.06	0.68 ± 0.06	0.67 ± 0.04	0.65 ± 0.04
	V2	0.74 ± 0.04	0.65 ± 0.06	0.70 ± 0.05	0.68 ± 0.04	0.67 ± 0.04
	V3	0.77 ± 0.04	0.69 ± 0.06	0.71 ± 0.06	0.70 ± 0.05	0.69 ± 0.05
XGBoost	V1	0.09 ± 0.04	0.62 ± 0.05	0.66 ± 0.06	0.65 ± 0.04	0.63 ± 0.04
	V2	0.74 ± 0.05	0.66 ± 0.07	0.69 ± 0.06	0.68 ± 0.05	0.67 ± 0.05
	V3	0.76 ± 0.04	0.67 ± 0.07	0.71 ± 0.05	0.70 ± 0.04	0.69 ± 0.04

* For V1, 57 features were used to train the model, 103 for V2, and 138 for V3. Detail of features used can be seen in supplement table 1-5.

**Table 2:** Detailed summary of early sPE vs NPH model performance per visit for RF

Visits	AUC	Sensitivity (TP/ TP+FN)	Specificity (TN/ FP + TN)	PPV (TP/ TP +FP)	NPV (TN/FN+TN)
**V1**	0.76 ± 0.09	0.71 ± 0.12	0.69 ± 0.12	0.70 ± 0.10	0.71 ± 0.10
**V2**	0.35 ± 0.07	0.86 ± 0.09	0.70 ± 0.12	0.75 ± 0.03	0.84 ± 0.09
**V3**	0.88 ± 0.07	0.89 ± 0.09	0.70 ± 0.14	0.76 ± 0.09	0.87 ± 0.10

* For V1, 57 features were used to train the model, 103 for V2, and 138 for V3. Detail of features used can be seen in supplement table 1-5.

**Table 3:** Detailed summary of early sPE vs late sPE+E model performance per visit for 4 classifiers

Model	Visits	AUC	Sensitivity (TP/TP+FN)	Specificity (TN/FP+TN)	PPV (TP/TP+FP)	NPV (TN/FN+TN)
**LR**	**V1**	0.56 ± 0.10	0.53 ± 0.14	0.58 ± 0.14	0.56 ± 0.11	0.55 ± 0.10
	**V2**	0.69 ± 0.10	0.62 ± 0.14	0.67 ± 0.14	0.66 ± 0.12	0.64 ± 0.11
	**V3**	0.75 ± 0.10	0.67 ± 0.14	0.71 ± 0.14	0.71 ± 0.11	0.69 ± 0.10
**SVM**	**V1**	0.59 ± 0.12	0.44 ± 0.15	0.70 ± 0.16	0.60 ± 0.16	0.55 ± 0.09
	**V2**	0.71 ± 0.09	0.48 ± 0.15	0.81 ± 0.12	0.73 ± 0.15	0.61 ± 0.08
	**V3**	0.77 ± 0.08	0.59 ± 0.12	0.88 ± 0.09	0.84 ± 0.11	0.69 ± 0.07
**RF**	**V1**	0.64 ± 0.11	0.61 ± 0.15	0.61 ± 0.15	0.62 ± 0.11	0.62 ± 0.11
	**V2**	0.76 ± 0.08	0.66 ± 0.14	0.71 ± 0.14	0.70 ± 0.11	0.68 ± 0.10
	**V3**	0.83 ± 0.08	0.74 ± 0.13	0.76 ± 0.13	0.76 ± 0.10	0.75 ± 0.11
**XGBoo** **st**	**V1**	0.62 ± 0.10	0.60 ± 0.10	0.57 ± 0.09	0.60 ± 0.11	0.59 ± 0.04
	**V2**	0.76 ± 0.09	0.43 ± 0.13	0.92 ± 0.08	0.85 ± 0.13	0.62 ± 0.06
	**V3**	0.82 ± 0.08	0.53 ± 0.15	0.91 ± 0.08	0.86 ± 0.12	0.67 ± 0.08

* For V1, 57 features were used to train the model, 103 for V2, and 138 for V3. Detail of features used can be seen in supplement table 1-5.

**Table 4:** Our model versus other models

	AUC	Sensitivity (TP/ TP+FN)	Specificity (TN/ FP + TN)	PPV (TP/ TP +FP)	NPV (TN/FN+TN)
**Poon et al.**	0.68 ± 0.04	0.62 ± 0.05	0.67 ± 0.07	0.65 ± 0.05	0.64 ± 0.04
**Akolerkar et al.**	0.69 ± 0.05	0.63 ± 0.07	0.67 ± 0.06	0.66 ± 0.04	0.65 ± 0.04
**PEPrML (Our Model)**	0.72 ± 0.04	0.64 ± 0.06	0.68 ± 0.06	0.67 ± 0.04	0.65 ± 0.04

* All models were evaluated at V1. Poon et al. utilized features derived from maternal risk factors, MAP, PlGF, uterine artery pulsatility index, and PAPP-A. On the other hand, Akolerkar et al. incorporated all the features used by Poon et al. as well as additional placental analytes features.
